# Trust your struggle

**DOI:** 10.1007/s40037-016-0259-3

**Published:** 2016-03-14

**Authors:** Pim W. Teunissen

**Affiliations:** Department of Educational Development and Research, School of Health Professions Education (SHE), Faculty of Health Medicine and Life Sciences, Maastricht University, Maastricht, The Netherlands; Department of Obstetrics and Gynaecology, VU University Medical Center, Amsterdam, The Netherlands

Transitions in medical education are challenging for students, postgraduate trainees, their preceptors, and everyone else in the workplace [1]. This issue of Perspectives on Medical Education features a paper by Atherley and colleagues that clearly illustrates these challenges for undergraduate medical students [2]. They focused their transition study on the shift of context that students experience within rotation-based clerkships when moving from one rotation to the next. Some interesting findings emerged. For instance, students recognized the need to start a new rotation with a positive attitude and to try and develop a good relationship with staff. Paradoxically, both intentions could require students to ignore reputations of a rotation and its people. If not ignored, anticipation could stand in the way of taking full advantage of the learning opportunities of a rotation [2]. Fortunately, many students reported that the struggle of working through a transition added to their professional identity development and led to increased confidence.

Besides contributing to our insight into transitions, Atherley and colleagues’ paper significantly contributes to medical education research in another way. It is a great example of theory-informed medical education research. The researchers chose organizational socialization theory as their framework to study student transitions. In their introduction they described the components provided by this theory: newcomer characteristics, behaviours, and organizational efforts, their influences on newcomer adjustments and ultimately the outcomes of the process of organizational socialization [3]. This conceptual framework was not forced upon the data. It was used dialectically to both inform data analysis and lead to theory refinement. In their discussion, Atherley and colleagues based their implications and recommendations on an adapted version of the organizational socialization model that they started with [2].

Theory-informed research in medical education can take many shapes, from the example referred to above to quantitative research where theory informs hypothesis testing or qualitative research where theory determines how phenomena should be interpreted [4, 5]. Theory provides researchers with ‘ways of thinking about a problem or a study, or ways of representing how complex things work the way they do’ [6]. More broadly speaking, it offers a framework of concepts to apply to the research problem under investigation. Bordage explained that conceptual frameworks originate from [6]:

- theories with well-organized principles and propositions that have been confirmed by observations or experiments;
- models derived from theories, observations or sets of concepts, or
- evidence-based best practices derived from outcome and effectiveness studies.

Although having a conceptual framework to guide your studies may seem a very welcome tool to medical education researchers, many struggled to use it in their work. For over 15 years, worries about the lack of explicitly mentioned conceptual frameworks in medical education research have been published in leading journals. In 2000, Prideaux and Spencer commented: ‘the advancement of medical education also requires that it draws on a strong theoretical base from the parent discipline of education’ [7]. In a critical examination of experimental studies in medical education published in 2003–2004, Cook and colleagues found that only 55 % presented a conceptual framework [8]. In 2010, two commentaries again had to conclude that there was still a lack of research that clearly describes how it was informed by and built on a conceptual framework [9, 10]. Now, in 2016, I believe it is fair to say that the field of medical education research seems to have taken this criticism seriously. The number of conceptual frameworks that could be relevant to medical education researchers appears to be increasing. Journals have dedicated special sections to papers that introduce concepts from fields such as psychology, sociology, anthropology, architecture, or neurosciences.

However, for individual researchers and the teams in which they work, engaging with conceptual frameworks will require continuous efforts. In my own experience and in working with (PhD) researchers and giving workshops on the topic, I found that recognizing there are many potentially relevant concepts surrounding one’s topic of research is both necessary and confusing. There is a parallel between medical students transitioning to a new rotation and medical education researchers engaging with (new) conceptual frameworks. Although you know there is much to learn from engaging with new ideas and concepts, it is also impossible to know at the start how you will manage to achieve a positive outcome while the possibility of ‘getting lost’ is looming.

In order to constructively engage with conceptual frameworks and to help others to do the same, I find it helpful to recognize the contributions of the preconditions: freedom and safety. Any researcher, certainly one who enters a new field of study, will have to find the personal and social freedom to challenge assumptions. Understanding why a research question is of interest to you or your team is usually not that difficult to answer. However, scientific research requires adding a piece of knowledge or insight to issues that transcend local problems that may have led to the research. One’s context should not be viewed as the reason for a study but as the practice in which a study can be conducted [11]. The research question should be grounded in one of the theories, models or previous studies that also form the origins of conceptual frameworks. This shift of perspective offers freedom to individual researchers and their teams to explore and subsequently choose from the multiple lines of reasoning that could underpin a research question. The freedom that comes with making these choices can lead to uncertainty: can I become familiar enough with unknown theories, models or previous research in order conduct this research, do I want to make the shift away from local problems as the reason for study? For these and other questions to be answered confirmatory, researchers need to feel safe. Safe enough to ask these questions and know that other team members will help explore new conceptual frameworks or help open up access to insightful sources of information or subject experts. Safe enough to make the shift away from local problems to using one’s context to add a little piece of knowledge to science. In the end, it is this process that will eventually help find more sophisticated answers to local problems. Just as in a transition, it takes trust in your struggle to achieve personal, and in this case scientific, development.

## Disclosers

No grants supported the preparation of this manuscript.

## References

[1] Teunissen PW, Westerman M (2011). Opportunity or threat: the ambiguity of the consequences of transitions in medical education. Med Educ.

[2] Atherley A, Hambleton I, Unwin N, George C, Lashley P, Taylor C. Exploring the transition of undergraduate medical students into a clinical clerkship using organisational socialisation theory. Perspect Med Educ. 2016;5 DOI: 10.1007/s40037-015-0241-5. 10.1007/s40037-015-0241-5 PMC4839013 26951164

[3] Bauer T, Erdogan B, Zedeck S (2011). Organizational socialisation: the effective onboarding of new employees. Maintaining, expanding, and contracting the organization: APA handbooks in psychology.

[4] Bergman EM, de Bruin ABH, Vorstenbosch MATM (2015). Effects of learning content in context on knowledge acquisition and recall: a pretest-posttest control group design. BMC Med Educ.

[5] Whitehead C, Selleger V, van de Kreeke J, Hodges B (2014). The ‘missing person’ in roles-based competency models: a historical, cross-national, contrastive case study. Med Educ.

[6] Bordage G (2009). Conceptual frameworks to illuminate and magnify. Med Educ.

[7] Prideaux D, Spencer J (2000). On theory in medical education. Med Educ.

[8] Cook DA, Beckman TJ, Bordage G (2007). Quality of reporting of experimental studies in medical education: a systematic review. Med Educ.

[9] Rees CE, Monrouxe LV (2010). Theory in medical education research: how do we get there?. Med Educ.

[10] Teunissen PW (2010). On the transfer of theory to the practice of research and education. Med Educ.

[11] Norman G (2014). Data dredging, salami-slicing, and other successful strategies to ensure rejection: twelve tips on how to not get your paper published. Adv Health Sci Educ Theory Pract.

